# The first composite score predicting Digital Ulcers in systemic sclerosis patients using Clinical data, Imaging and Patient history—CIP-DUS

**DOI:** 10.1186/s13075-020-02235-7

**Published:** 2020-06-15

**Authors:** S. Friedrich, S. Lüders, J. Klotsche, G. R. Burmester, G. Riemekasten, S. Ohrndorf

**Affiliations:** 1 Department of Rheumatology and Clinical Immunology, Charité – Universitätsmedizin Berlin, Berlin, Germany; 2 Department of Radiology, Charité – Universitätsmedizin Berlin, Berlin, Germany; 3 Department of Gastroenterology and Rheumatology, Charité – Universitätsmedizin Berlin, Berlin, Germany; 4 German Rheumatism Research Center, a Leibniz Institute, Berlin, Germany; 5 Institute for Social Medicine, Epidemiology and Health Economics, Charité - Universitätsmedizin Berlin, Berlin, Germany; 6 Department of Rheumatology and Clinical Immunology, University of Schleswig-Holstein, Lübeck, Germany

**Keywords:** Systemic sclerosis, Capillaroscopy, Colour Doppler ultrasound, Fluorescence optical imaging, Modified Rodnan skin score, Disturbed microcirculation, Digital ulcers

## Abstract

**Background:**

Digital ulcers (DU) present a challenging complication in systemic sclerosis (SSc). The aim of this study was to combine clinical characteristics and imaging methods to a composite score for the prediction of DU in SSc patients.

**Methods:**

Seventy-nine SSc patients received clinical examination, their patient history was taken and nailfold capillaroscopy (NC), colour Doppler ultrasonography (CDUS) and fluorescence optical imaging (FOI) of the hands were performed at baseline. Newly developed DU over a period of approximately 12 months were registered. We used criteria with area under the curve (AUC) of at least 0.6 in regard to the development of these new DU to create the score (CIP-DUS, clinical features, imaging, patient history—digital ulcer score).

**Results:**

Twenty-nine percent of all SSc patients developed new DU during follow-up (48.1% diffuse, 18.4% limited SSc). Based on the cross-validated (cv) AUC, a weight (cvAUC > 0.6 and ≤ 0.65: 1; cvAUC > 0.65 and ≤ 0.7: 2; cvAUC > 0.7: 3) was assigned to each selected parameter. The performance of the final CIP-DUS was assessed with and without the CDUS/FOI component. For the scleroderma patterns in NC, three points were appointed to *late*, two to *active* and one point to *early* capillaroscopy pattern according to Cutolo et al. The CIP-DUS including the CDUS and FOI parameters resulted in a good diagnostic performance (AUC after cross-validation: 0.83, 95%CI 0.74 to 0.92) and was well calibrated (chi-square = 12.3, *p* = 0.58). The cut-off associated with the maximum of sensitivity and specificity was estimated at ≥ 10 points resulting in a sensitivity of 100% and specificity of 74% for new DU during follow-up. Excluding CDUS and FOI parameters leads to a non-statistically significant lower performance (AUC after cross-validation: 0.81, 95%CI 0.72 to 0.91). However, including CDUS and FOI resulted in a better classification of patients in respect to the outcome new DU in follow-up due to significantly better reclassification performance (NRI = 62.1, *p* = 0.001) and discrimination improvement (IDI = 9.7, *p* = 0.01).

**Conclusion:**

A new score was introduced with the aim to predict digital ulcers. If applied correctly and with the new imaging techniques proposed, all patients at risk of digital ulcers throughout 12 months could be identified.

## Background

Digital ulcers (DU) affect more than a third of patients with systemic sclerosis (SSc) over time ensuing a considerable decrease in quality of life [1]. Different risk factors for the development of DU have already been described: male gender, history of digital ulcers, pathologic CSURI (Capillaroscopy Skin Ulcers Risk Index), and altered ESR (erythrocyte sedimentation rate) [2]. So far, limited efforts have been made to create a scoring system using capillaroscopic findings. First, CSURI was developed, which identifies patients at risk of developing new DU in a 3-month follow-up [3–5]. However, a multicentre study showed that 40% of their patients were not assessable with CSURI due to the absence of megacapillaries, which are an essential part of the index’s equation [6]. Furthermore, Caramaschi et al. developed a score of risk factors that was associated with ischemic digital ulcers in SSc patients undergoing Iloprost treatment. They identified four variables with high ORs for ischemic DU: age at disease onset < 47.0 years (OR 6.17), delay in beginning Iloprost therapy > 18 months (OR 5.70), history of smoking (OR 6.80), and presence of contractures (OR 6.50). Thus, they designed an additive model with one point per risk factor present. ROC analysis showed promising results with an AUC of 0.836 (95% CI 0.736–0.937) [7]. Manfredi et al. proposed a composite predictive model including “capillaroscopic, demographic and clinic-serological parameters” to identify patients at risk for new digital ulcers [2].

In other rheumatologic diseases, the use of composite scores is performed as a standard procedure, either to assess disease activity or severity, e.g. SLAM (Systemic Lupus Activity Measure), SLEDAI (Systemic Lupus Erythematosus Disease Activity Index) or BILAG (British Isles Lupus Assessment Group) for systemic lupus erythematosus; and DAS28 (Disease activity score 28), SDAI (simple disease activity index), and CDAI (clinical disease activity index) for rheumatoid arthritis, and many more in other indications such as psoriatic arthritis or ankylosing spondylitis. Most of these composite scores have been developed to reflect current disease activity, but also in order to account the emergence of new clinical manifestations of the underlying disease. Composite scores in the field of rheumatology usually take into account biomarkers or laboratory markers as well as clinical findings and information given by patients.

So far, there has not yet been presented a composite score created for patients with systemic sclerosis. Our aim was to create a composite score for the prediction of digital ulcers in SSc patients combining clinical data, imaging, and patient history based on the results that were recently published by our group [8].

## Patients and methods

Seventy-nine in- and out-patients with SSc were recruited. A signed informed consent was obtained from all patients. Three patients dropped out during the follow-up period: one patient withdrew her consent; two patients unfortunately died. After a medium of 12 months, we asked for newly developed digital ulcers during the follow-up period.

The included SSc patients received clinical examination, nailfold capillaroscopy (NC), colour Doppler ultrasonography (CDUS) [8, 9], and fluorescence optical imaging (FOI) [8, 10] of both hands at baseline, and also their patient history was taken as described in detail in our previous publications [8–10]. The two examiners were not blinded to patient history or clinical manifestations of the disease when performing the imaging techniques. Furthermore, NC was performed to assess for capillary density and capillaroscopy patterns (*late*, *active*, and *early*) introduced by Cutolo et al. in the year 2000.

### Statistical analyses

The association of each potential score component with the likelihood of newly developed DU was estimated by means of a logistic regression model. The area under curve (AUC) was determined to assess the predictive performance of each predictor variable. *K*-fold (*k* = 10) cross-validation was used to evaluate the predictive performance of each parameter to generate a more realistic estimate of predictive performance [11]. The idea of cross-validation is to randomly divide the data into *k* equal-sized parts. The *k*th part is left out, the model is fit to the other *k* − 1 parts, and then predictions for the left-out *k*th part are obtained. A parameter was included into the score if the resulting AUC reached a value of at least 0.6. Based on the cross-validated (cv) AUC, a weight (cvAUC > 0.6 and ≤ 0.65: 1; cvAUC > 0.65 and ≤ 0.7: 2; cvAUC > 0.7: 3) was assigned to each selected parameter. The performance of the final CIP-DUS was assessed with and without the CDUS/FOI component. The improved model performance by adding the CDUS/FOI component was evaluated by comparing the AUC using the algorithm suggested by DeLong [12]. In addition, the net reclassification improvement (NRI) and integrated discrimination improvement (IDI) [13] were calculated to evaluate the appropriate or inappropriate reclassification of patients after adding CDUS/FOI. Finally, the calibration of the logistic model was tested by calculating Hosmer-Lemeshow chi-square test. Statistical analyses were performed with STATA 12.1 (StataCorp. 2011. Stata Statistical Software: Release 12. College Station, TX: StataCorp LP).

## Results

Of the 76 SSc patients (62 female patients, *n* = 43 with limited cutaneous SSc and *n* = 19 with diffuse cutaneous SSC) that were questioned at 12 months follow-up, 29% reported newly developed DU (48.1% of patients with diffuse SSc and 18.4% of patients with limited SSc) (see Supplementary Table and [8]). The obtained AUC were calculated for potential parameters to be included in the CIP-DUS after *k*-fold cross-validation. A strong association with newly developed DU during follow-up was observed for history of digital ulcer and/or pitting scars (OR 36.2; 95% CI 2.1 to 626.9; AUC 0.77), present digital ulcers and/or pitting scars at baseline (OR 15.7; 95%CI 3.3 to 74.3; AUC 0.76), and the NC pattern (OR 18.6; 95%CI 1.1 to 326.4; AUC 0.70). The modified Rodnan skin score was moderately associated with the likelihood for newly developed ulcers at follow-up. A weight of one was assigned to the SSc subtype, pulmonary arterial hypertension, reduced capillary density (*n* < 7/mm) in digit III of the right hand in NC, missing initial enhancement in FOI in digit III of the right hand, and percentage of pathologic (i.e. narrowed or occluded) vessels > 35% in CDUS based on the AUC. For the scleroderma patterns in nailfold capillaroscopy as introduced by Cutolo et al., three points were appointed to *late*, two to *active*, and one to *early* capillaroscopy pattern (see Table 1).

**Table 1:** Clinical data, imaging and patient history used for the creation of the score (CIP-DUS)

	Feature	OR (95% CI)	***P*** value	Sensitivity*	Specificity*	AUC/cvAUC	Weight
**Clinical data and patient history**	Smoker at baseline	OR 2.4 (0.6–8.7)	0.28	22.7	88.9	–	**–**
	SSc diffuse subtype	OR 4.1 (1.5–11.7)	0.01	59.1%	74.1%	0.67/0.65	**1**
	Modified Rodnan skin score > 8	OR 9.4 (3.0–29.2)	< 0.01	68.2%	81.5%	0.75/0.73	**2**
	Pulmonary arterial hypertension	OR 4.7 (1.2–18.7)	0.03	27.3%	92.6%	0.56/0.54	**1**
	Present digital ulcers or pitting scars at baseline	OR 15.7 (3.3–74.3)	< 0.01	90.9%	61.1%	0.76/0.76	**3**
	History of digital ulcer or pitting scars	OR 36.2 (2.1–626.9)	< 0.01	100.0%	44.4%	0.78/0.77	**3**
**NC**	NC pattern (defined by Cutolo et al. 2000)	OR 18.6 (1.1–326.4)	< 0.01	100.0%	28.9%		
	- *Early*					0.58/0.55	**1**
	- *Active*					0.69/0.67	**2**
	- *Late*					0.78/0.76	**3**
	Late NC pattern vs. other NC patterns	OR 1.476 (0.5–4.0)	0.46	50.0%	59.6%	0.56/0.55	**–**
	Reduced capillary density (*n* < 7/mm) in digit III of the right hand in NC	OR 9.0 (1.1–73.6)	0.03	94.7%	33.3%	0.62/0.61	**1**
**FOI**	Missing initial enhancement in FOI in digit III of the right hand	OR 3.9 (1.2–12.8)	0.03	56.3%	75.0%	0.63/0.61	**1**
	Missing initial enhancement in FOI in ≥ 1 digit	OR 0.9 (0.3–2.8)	1.00 (ns)	62.5%	34.1%	–	**–**
	Complete disruption in FOI in ≥ 1 digit	OR 1.3 (0.3–5.9)	0.70 (ns)	25.0%	84.1%	–	**–**
**CDUS**	Percentage of pathologic (i.e. narrowed or occluded) vessels > 35% in CDUS	OR 4.3 (1.5–12.4)	0.01	68.2%	66. 7%	0.67/0.64	**1**
	≥ 1 pathologic vessel in CDUS in in digit III of the right hand	OR 3.8 (1.0–14.6)	0.05	90.9	28.3	–	**–**

*AUC* area under curve estimated in the source sample, *cvAUC* area under curve estimated by *k*-fold cross-validation, *OR* odds ratio, *CI* confidence interval, *NC* nailfold capillaroscopy, *FOI* fluorescence optical imaging, *CDUS* colour Doppler ultrasound

*Sensitivity and specificity were only calculated for parameters included in the CIP-DUS

**Fig. 1:**
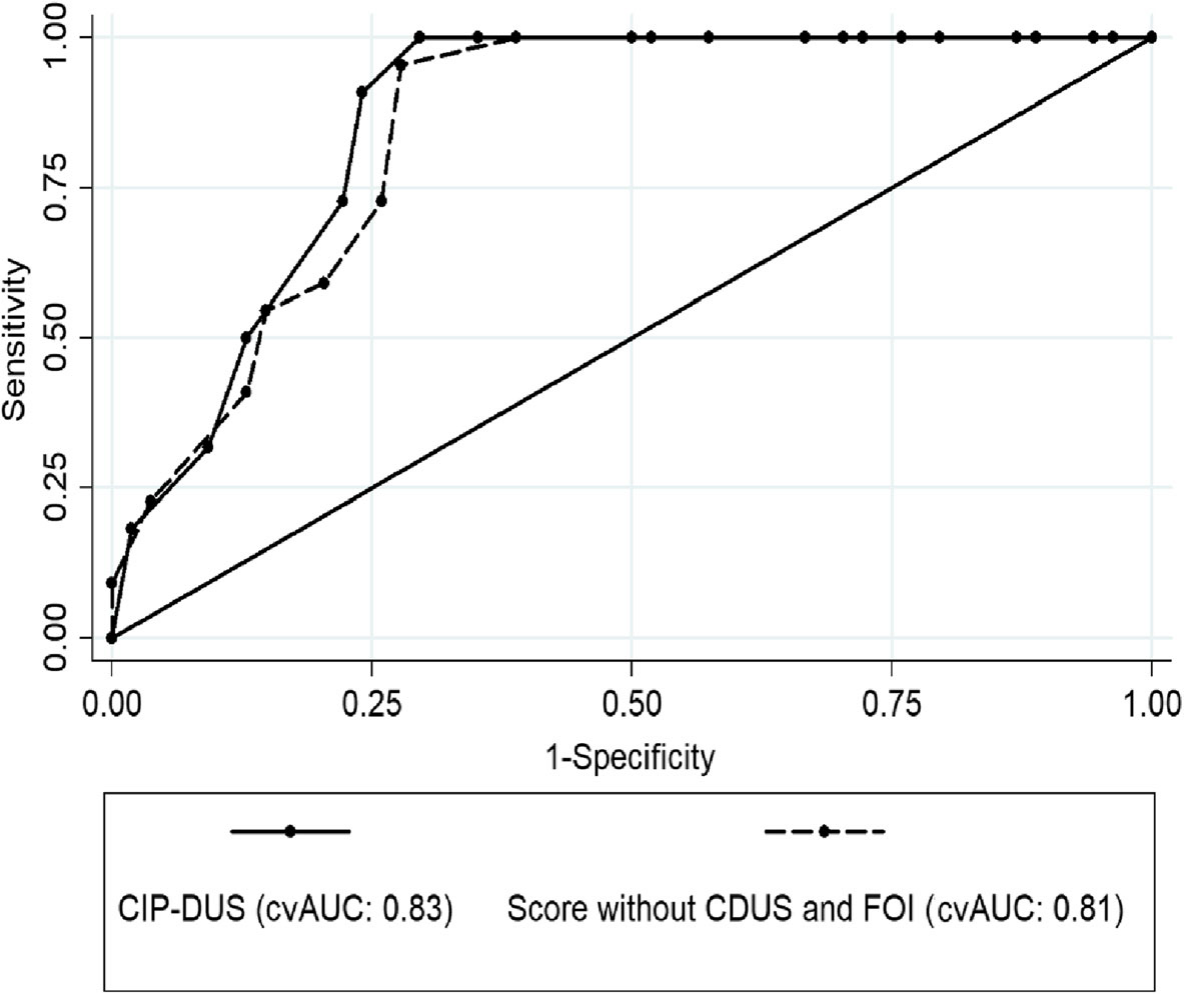
Receiver operating characteristic (ROC) of the CIP-DUS and the score in absence of CDUS and FOI regarding the development of new digital ulcers in a 12-month follow-up

The CIP-DUS including the CDUS and FOI parameters resulted in a good diagnostic performance (AUC after cross-validation: 0.83, 95%CI 0.74 to 0.92, see Fig. 1) and was well calibrated (chi-square = 12.3, *p* = 0.58). The cut-off associated with the maximum of sensitivity and specificity was estimated at ≥ 10 points resulting in a sensitivity of 100% and specificity of 74% for new DU during follow-up.

Alternatively, the score was calculated without inclusion of the CDUS and FOI parameters. It resulted in a slightly lower diagnostic performance (AUC after cross-validation: 0.81, 95%CI 0.72 to 0.91) as compared to CIP-DUS. The difference was not statistically significant (chi-square = 1.6, *p* = 0.20, Fig. 1). But the inclusion of CDUS and FOI resulted in a significantly better reclassification performance (NRI = 62.1, *p* = 0.001) and discrimination improvement (IDI = 9.7, *p* = 0.01) in comparison to the score in absence of CDUS and FOI indicating a better classification of patients in respect to the outcome DU at follow-up. In more detail, 14 (64% of 22 patients with DU at follow-up) had a higher predicted probability for a positive outcome by including CDUS and FOI, whereas 18 patients (33% of 54 patients without DU at follow-up) had a lower predicted probability for a positive outcome. The other way around, only 4 patients (18%) were assigned to a lower risk and 9 patients (18%) to a higher risk in patients with and without a DU at follow-up, respectively. It means that CIP-DUS (including CDUS and FOI) tended to result in higher predicted probabilities in patients with DU at follow-up and lower predicted probabilities in patients without, indicating a better diagnostic performance (sensitivity and specificity).

## Discussion

The aim of our project was to combine patient history, clinical and imaging findings in order to create a composite score able to determine the individual risk for the development of new DU in SSc patients during a 12-month follow-up. The odds ratios of known risk factors and other features were obtained.

Consistent with previous literature, current or previous digital ulcers and/or pitting scars were important factors in the recurrence of this complication [2, 14]. Our data also suggest a link between skin involvement (represented by a diffuse cutaneous subtype or a high mRSS) and the DU development [8]. Sunderkötter et al. [14] also found that a diffuse skin sclerosis significantly impacted on the appearance of DU in multivariate analysis, but only when pulmonary arterial hypertension was present. Here, in univariate analysis, both the diffuse subtype and pulmonary arterial hypertension (PAH) were associated with digital ulcers in a 12-month follow-up.

While PAH as a sign of systemic vasculopathy already links to the pathophysiology of digital ulcers, the evidence of micro- and macrovasculopathy in imaging is an established tool in the diagnostic and risk stratification of SSc patients. In accordance with our results, Smith et al. [15] found a more severe capillaroscopic pattern predictive for future severe peripheral vascular involvement. In the CAP study, the mean number of capillaries per millimetre in the middle finger of the dominant hand was identified as one of the strongest risk factors for new DU [16]. This is comparable with our finding that a reduced capillary density in the right middle finger indicated a higher risk of ulcers. Interestingly, pathologic findings in this finger detected by FOI and CDUS (not significant) were also associated with our primary end point. In CDUS however, an overall high percentage of pathologic vessels showed better predictive values.

While the single identified risk factors showed either low sensitivity (skin involvement, CDUS, FOI) or low specificity (DU presence/history, capillaroscopy) levels (data not shown), we aimed to combine the factors to a predictive score. The final composite score proved good diagnostic performance with perfect sensitivity of 100% and good specificity of 74% at a cut-off of ≥ 10 points. Excluding the uncommon imaging techniques CDUS and FOI, the score showed a slightly lower diagnostic performance in comparison to the complete CIP-DUS, which was not statistically significant. However, reclassification performance and discrimination improvement were significantly better when the full CIP-DUS (including CDUS and FOI) was assessed.

One limitation of the proposed CIP-DUS is the limited availability of the suggested imaging methods CDUS and FOI. Nevertheless, our data revealed an only slight decrease of the score’s performance when excluding those two imaging methods. This shows that with the commonly obtained information (clinical data, NC, patient history), which every clinician treating SSc patients should possess, a practical and powerful score assessment of the risk to develop new DU is still possible and should be integrated in clinical routine.

The assessment of DU is a challenging task. Occurrence of DU during follow-up was assessed via a telephone or personal interview and patients were instructed (using pictures) at baseline to look out for and document newly formed ulcers on their hands in order to improve accuracy at follow-up. A limitation of this study is the insufficient differentiation made between ischemic and traumatic ulcers.

Advantages of this study include the relatively long follow-up period of 12 months and the thorough characterisation of the patients including demographic, clinical, laboratory, and imaging data. Due to the complex pathophysiology of systemic sclerosis and its complications such as DU, taking all these factors into account is paramount when trying to reduce the risk of DU in the future.

As this is a pilot project, the predictive values of the composite score have unfortunately not yet been applied on an independent cohort. This should be conducted in future studies on a larger scale of patients—if only in the reduced form without FOI and CDUS. Even the reduced CIP-DUS, which integrates the usually present patient information about clinical presentation, patient history as well as NC pattern and capillary density can provide a powerful risk assessment for future DU in SSc patients.

## Conclusions

The new composite score CIP-DUS predicts digital ulcer development throughout a 12-month follow-up and identifies all “at risk” patients. Even without the new imaging features, which might not be available to all clinicians, the score still shows good performance in regard to detecting patients at risk for new DU development.

## Supplementary information

**Additional file 1:.** Supplement Table. Baseline patient characteristics, including diagnosis, sex, age (±SD), Raynaud’s phenomenon, digital ulcers and nailfold capillaroscopy patterns as described by Cutolo et al. (table cited from [8]).

## Data Availability

The datasets used and/or analysed during the current study are available from the corresponding author on reasonable request.

## Abbreviations

95%CI: 95% confidence interval
ACR/EUSTAR: The American College of Rheumatology/European League Against Rheumatism
AUC: Area under the curve
cvAUC: Cross-validated area under the curve
BILAG: British Isles Lupus Assessment Group
CDA: Clinical disease activity index
CDUS: Colour Doppler ultrasonography
CIP-DUS: Clinical data, imaging, patient history—digital ulcer score
CSURI: Capillaroscopy Skin Ulcers Risk Index
DAS28: Disease activity score 28
DU: Digital ulcers
FOI: Fluorescence optical imaging
ESR: Erythrocyte sedimentation rate
ICG: Indocyanine green
mRSS: Modified Rodnan skin score
OR: Odds ratio
PAH: Pulmonary arterial hypertension
ROC: Receiver operating characteristic
RP: Raynaud’s phenomenon
SSc: Systemic sclerosis
SDAI: Simple disease activity index
SLAM: Systemic Lupus Activity Measure
SLEDAI: Systemic Lupus Erythematosus Disease Activity Index
