# Supplementary material for: The first composite score predicting Digital Ulcers in systemic sclerosis patients using Clinical data, Imaging and Patient history—CIP-DUS
Source: Arthritis Res Ther. 2020 Jun 15;22:144. doi: 10.1186/s13075-020-02235-7 (PMC7294661; doi:10.1186/s13075-020-02235-7)
Supplement: Supplementary file 1 — Additional file 1:. Supplement Table. Baseline patient characteristics, including diagnosis, sex, age (±SD), Raynaud’s phenomenon, digital ulcers and nailfold capillaroscopy patterns as described by Cutolo et al. (table cited from [8]). [file 13075_2020_2235_MOESM1_ESM.docx]

***Supplement Table:*** *Baseline patient characteristics, including diagnosis, sex, age (± SD), Raynaud’s phenomenon, digital ulcers and nailfold capillaroscopy patterns as described by Cutolo et al. (table cited from [8])*

| **Patient characteristics** | **Systemic sclerosis (SSc**) | Limited (cutaneous) SSc | Diffuse cutaneous SSc |  |
| --- | --- | --- | --- | --- |
|  | n=76 | n=49 | n=27 | P-value |
| ♀**: n (%)** | 62 (81.6%) | 43 (87.8%) | 19 (70.4%) | ns |
| **Mean age in years**  **(±SD)** | 56.0 (±14.3) | 57.6 (±14.7) | 53.0 (±13.4) | ns |
| **Smokers** | 11 (14.5%) | 4 (8.2%) | 7 (25.9%) | 0.0461 |
| **Mean disease duration (in years ±SD) since**  First Raynaud’s symptoms  First non-Raynaud’s symptoms | 13.4 (±12.6)  9.6 (±8.7) | 14.5 (±12.4)  9.6 (±8.8) | 11.4 (±13.0)  9.7 (±8.7) | ns  ns |
| **Raynaud’s phenomenon,**  **n (%)** | 70 (92.1%) | 45 (91.8%) | 25 (92.6%) | ns |
| **History of digital ulcers,**  **n (%)** | 36 (47.4%) | 18 (36.7%)  * | 18 (66.7%) | 0.0167 |
| **Mean modified Rodnan skin score (±SD)** | 8.6 (±8.2) | 4.5 (±3.3)  *** | 16.2 (±9.1) | <0.0001 |
| **Organ involvement, n (%)**  - Lung  - Gastrointestinal  - Heart | 30 (39.5%)  51 (67.1%)  11 (14.5%) | 13 (26.5%)  30 (61.2%)  8 (16.3%) | 17 (62.9%)  21 (77.8%)  3 (11.1%) | 0.0030  ns  ns |
| **Antibodies**  ACA-positive  Scl70-positive | 25 (32.9%)  31 (40.7%) | **  22 (44.9%)  *  15 (30.6%) | 3 (11.1%)  16 (59.3%) | 0.0044  0.0272 |
| **Current medications**  - Iloprost  - Bosentan  - Oral vasodilator drugs* | 43 (56.6%)  11 (14.5%)  50 (65.8%) | 29 (59.2%)  6 (12.2%)  29 (59.2%) | 14 (51.8%)  5 (18.5%)  21 (77.8%) | ns  ns  ns |
| **Capillaroscopic pattern:** - Early | 13 (17.1%) | *  11 (22.4%) | 2 (7.4%) |  |
| - Active | 27 (35.5%) | 19 (38.7%) | 8 (29.6%) | 0.0341 |
| - Late | 32 (42.1%) | 15 (30.6%) | 17 (62.9%) |  |
| - Non-SSc | 2 (2.6%) | 2 (4.1%) | 0 |  |
| - Not determined | 2 (2.6%) | 2 (4.1%) | 0 |  |
| * ACE inhibitors, AT1 blockers, PDE5 inhibitors, calcium channel blockers, alpha1 antagonists | | | | |
